# Simultaneous Measurement of Refractive Index and Temperature Based on SMF–HCF–FCF–HCF–SMF Fiber Structure

**DOI:** 10.3390/s22228897

**Published:** 2022-11-17

**Authors:** Ronghui Xu, Chengran Ke, Yipu Xue, Yifei Xu, Minmin Xue, Jingfu Ye, Houquan Liu, Ming Chen, Shiliang Qu, Libo Yuan

**Affiliations:** 1 Photonics Research Center, School of Optoelectronic Engineering, Guilin University of Electronic Technology, Guilin 541004, China; 2 Guangxi Key Laboratory of Optoelectronic Information Processing, Guilin University of Electronics Technology, Guilin 541004, China

**Keywords:** multi-mode interference, optical fiber dual-parameter sensor, simultaneous measurement

## Abstract

In this research, we proposed and experimentally verified a compact all-fiber sensor that can measure refractive index (RI) and temperature simultaneously. Two segments of hollow-core fiber (HCF) are connected to the two ends of the four-core fiber (FCF) as a beam splitter and a coupler, and then spliced with two sections of single-mode fibers (lead-in and lead-out SMF), respectively. The two hollow-core fibers can excite the higher-order modes of the four-core fiber and recouple the core modes and higher-order modes into the outgoing single-mode fiber, thereby forming inter-mode interference. The different response sensitivities of two interference dips to RI and temperature manifest that the proposed structure can achieve simultaneous measurement. From the experimental results, it can be seen that the maximum sensitivity of the sensor to RI and temperature is 275.30 nm/RIU and 94.4 pm/°C, respectively. When the wavelength resolution is 0.02 nm, the RI and temperature resolutions of the sensor are 7.74 × 10^−5^ RIU and 0.335 °C. The proposed dual-parameter optical sensor has the advantages of high sensitivities, good repeatability, simple fabrication, and structure. In addition, it has potential application value in multi-parameter simultaneous measurement.

## 1. Introduction

Sensing technology research is important in many areas, including biomedicine, automotive industries, chemical industry, aerospace, geological exploration, petroleum storage, and so on [1]. Traditional sensors are often electronic devices made of metal materials, which are susceptible to environmental factors such as electromagnetic interference and chemical corrosion. Optical fiber sensors attracted much attention because of their small size, good performance, anti electromagnetic interference, high sensitivity and other unique advantages [2,3]. In many applications, the refractive index (RI) is a common and important sensing parameter. The optical fiber RI sensor is playing an increasingly important role in many application fields [4]. At present, many sensing structures can be used to measure refractive index, such as Mach–Zehnder interferometer (MZI) [5,6], Fabry–Perot interferometer (FPI) [7,8,9,10], fiber Bragg grating (FBG) [11,12,13], and long-period fiber grating (LPFG) [14,15,16]. Obviously, various RI sensors based on optical MZI were widely researched because of their high sensitivity and simple fabrication. For the past few years, more and more research was done on RI sensors based on multipath fiber MZI. Weihs et al. [17] proved that compared with the conventional double-path interferometer, the multi-path fiber MZI based on the interference between different core transmission modes has higher phase change sensitivity [18,19]. The multicore optical fiber (MCF) is sensitive to physical parameters and easy to splice, and is used as a multipath MZI (m-MZI). The commonly used multicore fibers include two-core fiber [20,21], three-core fiber [22], four-core fiber [23], seven-core fiber [24] and so on.

Additionally, the RI value is closely related to the temperature. To improve the RI measurement accuracy, temperature and RI need to be measured simultaneously, which not only avoids the additional errors caused by cross-sensitivity but also simplifies the temperature compensation system. At present, many optical fiber sensors for simultaneous measurement of RI and temperature are reported. Fiber gratings, such as LPFG and FBG, because of their temperature-sensitive but not RI-sensitive properties, are usually cascaded with other sensing structures to measure RI and temperature simultaneously [25,26,27,28]. In 2014, Bai et al. [29] demonstrated a compact all-fiber sensor based on a no-core fiber (NCF) and FBG. The NCF–FBG–NCF (NFN) structure is formed by splicing an FBG between two NCF segments, where the NCF acts as a beam splitter and coupler. The experimental results show that in the refractive index range of 1.333 to 1.398, the sensitivity is 109.573 nm/RIU, and in the temperature range of 10 °C to 70 °C, the sensitivity is 0.014 nm/°C. Cao et al. [30] proposed a fiber sensor by cascading a FBG with a peanut-shape structure and a core-offset structure. These two structures are formed by single-mode fibers through different fusion splicing techniques. The maximum RI sensitivity and temperature sensitivity are −26.965 nm/RIU and 0.08329 nm/°C, respectively. In 2021, Siti Mahfuza, Saimon et al. [31] obtained a novel optical fiber sensor that can simultaneously measure temperature and high refractive index by cascading a single-mode silicon rod single-mode fiber structure on a FBG. By monitoring the output power and wavelength shift of the transmission spectrum, it can be obtained that in the RI range of 1.45 RIU to 1.531 RIU, the sensitivity is 108.07 dBm/RIU, and from 35 °C to 85 °C, the temperature sensitivity is 9.31 pm/°C. In order to realize large-scale production and consider the development of practical applications, the structure of the optical fiber interferometer with a simple manufacturing process is of great value for simultaneous measurement.

In this work, we propose an all-fiber sensor based on the single-mode fiber–hollow-core fiber–four-core fiber–hollow-core fiber–single-mode fiber (SMF–HCF–FCF–HCF–SMF) structure obtained by direct fusion splicing technology, which is very simple to fabricate because only fusion splicing is involved. While the HCFs act as a beam splitter and optical coupler to excite high-order modes, and multipath MZI can be easily implemented with FCF. For the proposed sensor, the maximum fringe visibility of interference resonance dips can reach 24 dB. Temperature and RI can be measured simultaneously by monitoring the wavelength changes of two dips. The sensor has the advantages of being all-fiber, easy to manufacture, a compact size, etc.

## 2. Structural Design and Working Principle

**Figure 1:**
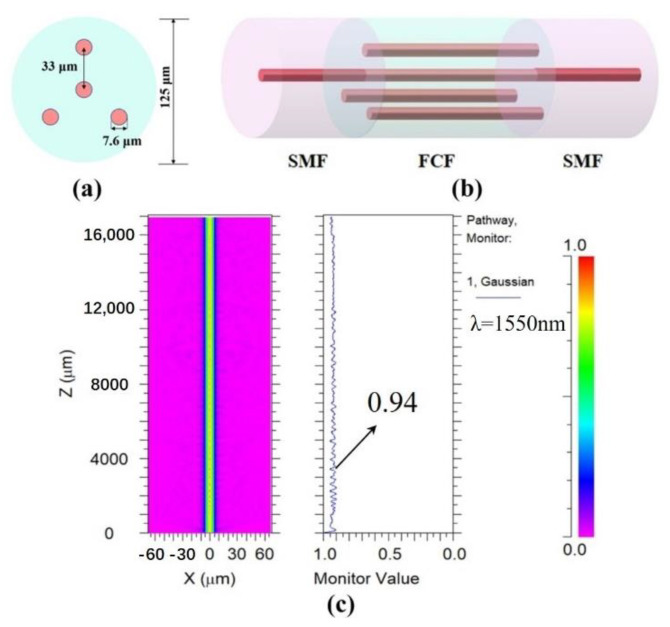
(**a**) The cross section of FCF; (**b**) the schematic diagram of the SMF–FCF–SMF fiber structure; (**c**) Simulated light field distribution of SMF–FCF–SMF structure.

The hollow core diameter of HCF used in this experiment is 10 μm, and the cladding diameter is 125 μm. Figure 1a shows a cross section of the FCF. The cores of the FCF are distributed in an equilateral triangle. One core is located at the center of the triangle, and the other three cores are located at the three vertices of the triangle, respectively. The distance from the surrounding cores to the middle core is 33 μm, each core has the same diameter of 7.6 μm, and the diameter of the cladding is about 125 μm. The schematic diagram of the structure when FCF is fused directly with two sections of SMF is shown in Figure 1b. The light field distribution is simulated, as shown in Figure 1c. From the figure, we can see that when the FCF is directly fused with input SMF and output SMF, the light field intensity is mainly distributed in the central core and cannot form an interferometer because the central core of the FCF is the same diameter as the core of the SMF and does not excite high-order modes.

**Figure 2:**
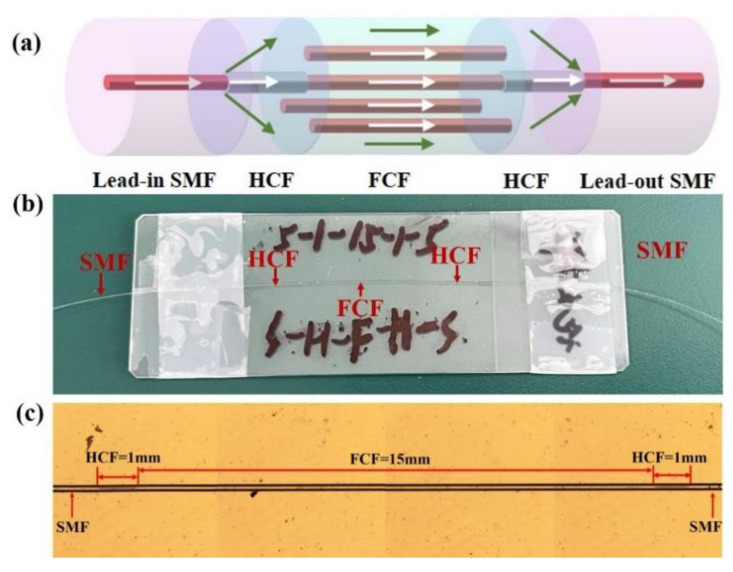
(**a**) The schematic diagram of the proposed SMF–HCF–FCF–HCF–SMF structure; (**b**) the photo of the actual structure; and (**c**) the photograph under the microscope.

**Figure 3:**
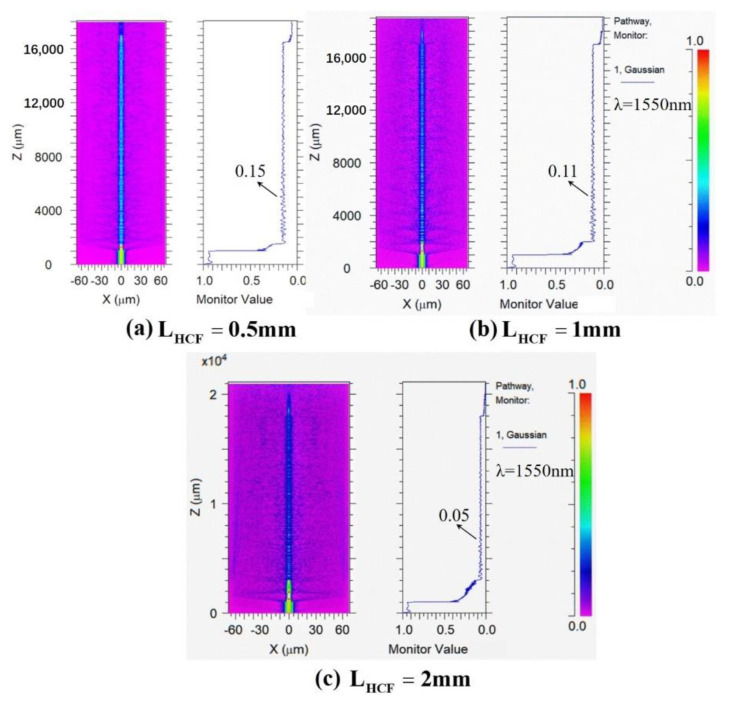
(**a**–**c**) Light-field distribution simulation with different HCF lengths.

To excite the high-order modes, we splice the FCF between two segments of HCF by a fusion splicer, and then splice the two SMFs at the other end of the two HCFs to form the SMF–HCF–FCF–HCF–SMF structure. The fabrication of the sensor structure only involves the fusion of optical fibers. The steps are as follows: First, in order to ensure that the sensing area can better perceive the changes of external factors, the surface coatings of SMF, HCF, and FCF need to be peeled off before the fusion, and wiped with alcohol to keep them clean and tidy. In order to achieve accurate cutting of the fiber length, we use a fixed-length cutting system in the process of making samples, which is capable of cutting with micron-level accuracy. Then we put the cut optical fibers into the fiber fusion splicer (S178C, Furukawa Electric, Tokyo, Japan), and adjust the fiber fusion splicer to “automatic welding mode”. After the discharge intensity and discharge time are set to 90 bits and 1300 ms respectively, the fusion splicer can recognize, align, and splice these different fibers automatically. At the same time, for the accuracy of the experiment, the control loss during splicing is less than 0.03 dB. The schematic diagram, the photo of the actual structure and the photograph under the microscope of the proposed sensor based on the SMF–HCF–FCF–HCF–SMF structure are shown in Figure 2a–c, respectively. For the proposed sensor, the two HCFs are used as a beam splitter and combiner, and the two SMFs act as lead-in and lead-out fibers. The light field propagation and intensity distribution of the SMF–HCF–FCF–HCF–SMF structure simulated by using the RSOFT software package with the finite difference beam propagation method (FD-BPM). As shown in Figure 3a–c, in the simulation process, we set all the incident light to 1550 nm, and the length of the input and output SMF is 1 mm. The length of FCF remains unchanged at 15 mm and the length of HCFs are regulated to 0.5 mm, 1 mm, and 2 mm, respectively. After inputting the effective refractive indices of the cladding and core, the software can obtain the optical field distribution of the whole waveguide by using FD-BPM. From Figure 3a–c, it can be seen that when HCF is added, the intensity of the light field is distributed not only in the central core but also in the other cores and cladding of the FCF. The HCF acts as a beam splitter and light can be coupled from the SMF into all cores of the FCF. Moreover, we can see that with the increase in HCF length, the coupling efficiency and interference strength gradually increases, but the insertion loss also increases at the same time. Considering these factors, we choose an HCF length of 1 mm.

When the incident light from the lead-in SMF passes through the first section of the HCF, different high-order modes are excited due to the mode field mismatch between the SMF and HCF, and the diameter of the effective mode field increases. Then, the HCF acts as a coupler, coupling the light into the four cores of the FCF at the first HCF–FCF interface, and coupling a portion of the energy into the cladding of the FCF to excite multiple high-order modes (four-core mode and cladding mode) of the FCF. Multiple modes propagate along the FCF and meet in the second HCF. Multiple phase differences along different light paths produce multi-path interference and form multipath MZI. The interference signal is coupled through the second HCF to the core of the lead SMF. The output spectrum will be the superposition of multiple interference patterns [32]. The output intensity of interference fringes can be written by the following equation [33]:

(1) $$I=I_{1}+I_{2}+2\sqrt{I_{1}I_{2}}\cos\phi$$

where *I* is the total light intensity, *I*_1_ and *I*_2_ are the light intensities of the core mode and the coupled cladding mode, respectively, ϕ is the phase difference between the modes, which can be expressed as:

(2) $$\phi =\frac{2\pi \Delta n_{eff}L}{\lambda }$$

where λ is the wavelength of the incident light, $\Delta n_{eff}$ is the effective refractive index difference between the modes involved in the interference, and L is the length of the FCF. If the phase difference satisfies the condition ϕ = (2*m* + 1) *π*, where m = 1,2,3…, the wavelength of the interference valley can be expressed as:

(3) $$\lambda_{m}=\frac{2\Delta n_{eff}L}{2m+1}$$

As can be seen from Equation (2), the change in $\Delta n_{eff}$ or *L* will affect the shift of wavelength. In addition, these changes are also affected by environmental changes such as RI, curvature, temperature, etc. Therefore, it is possible to measure these external physical parameters by monitoring the wavelength drift of specific interference valleys. When the external RI changes, the wavelength shift can be expressed as [34]:

(4) $$\Delta \lambda_{m}=\frac{2(\Delta n_{eff}+\Delta n)L}{2m+1}-\frac{2\Delta n_{eff}L}{2m+1}=\frac{2\Delta nL}{2m+1}$$

where Δn is the change in $\Delta n_{eff}$ with external RI. When the temperature is applied to the sensor, the shift of $\Delta \lambda_{m}$ can be described as:

(5) $$\frac{\Delta \lambda_{m}}{\lambda_{m}}=(\alpha +\zeta )\Delta T=(\alpha +\frac{\zeta_{1}^{}n_{1}^{}-\zeta_{2}n_{2}}{n_{1}^{}-n_{2}^{}})\Delta T$$

where α is the thermal expansion coefficient of the fiber, $\zeta_{1}$ and $\zeta_{2}$ are the thermo-optical coefficients of the two modes involved in the interference, respectively. $n_{1}$ and $n_{2}$ are the effective refractive index of the two modes. For the characteristics of optical fiber, the influence of thermal expansion’s effect on the temperature sensitivity of optical fiber is much less than that of the thermo-optical effect. Therefore, the temperature sensitivity mainly depends on the thermo-optical effect [35]. We can see that due to the different thermo-optical coefficients of different modes, ζ can be positive or negative, meaning that the interference wavelength may be red-shifted or blue-shifted as the temperature changes.

## 3. Experimental Results and Discussion

### 3.1. Intermodal Interference Spectrum of Sensor

**Figure 4:**
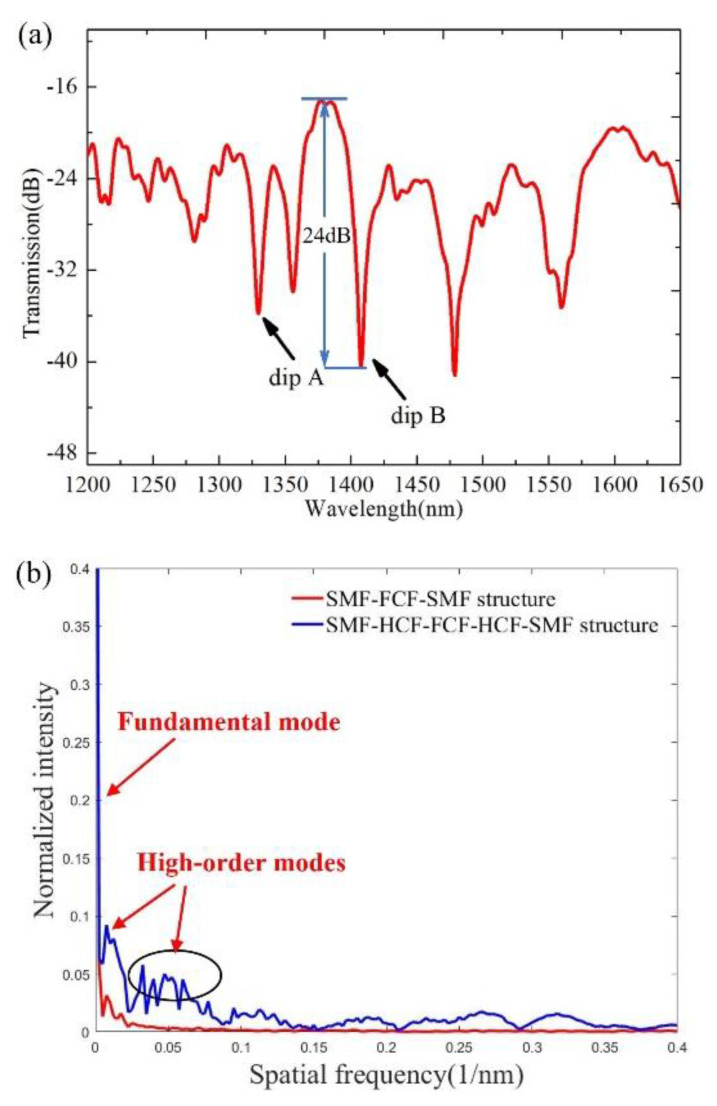
(**a**) Measured transmission spectrum of the sensor head in the air at room temperature. (**b**) Spatial frequency spectra of the proposed SMF–HCF–FCF–HCF–SMF structure with and without HCFs inserted.

Figure 4a shows a schematic of the transmission spectrum of the sensor in air at room temperature. It can be seen that we can observe the interference fringes with good visibility in the spectral range of 1200–1650 nm. The maximum fringe visibility of the interference resonance dips can reach 24 dB. Additionally, the transmission spectra of the proposed SMF–HCF–FCF–HCF–SMF structure and the SMF–FCF–SMF structure are subjected to fast Fourier transform (FFT) to analyze the number and power distribution of interference modes. The obtained spatial spectrum is shown in Figure 4b. It can be seen that no other modes are excited when the FCF is fused directly with the SMFs, which is well in agreement with the simulation result in Figure 1c. For the proposed SMF–HCF–FCF–HCF–SMF structure with a 15 mm length of FCF, it can be seen that in addition to one peak with higher intensity, there are many peaks with lower intensity. This indicates that the spectrum of the sensor is dominated by the interference occurring in the core mode and the cladding mode, but there are still some other weak interference modes, such as the interference between the central core mode and the surrounding core modes, and the interference between the surrounding core modes and the cladding mode. Therefore, there are multiple higher-order modes involved in the interference, and the interference spectrum is a combination of multiple modes. To measure two different parameters, RI and temperature changes will be applied to the sensor, respectively. In this experiment, dip A and dip B are chosen as the sensing dips.

### 3.2. Refractive Index Sensing

**Figure 5:**
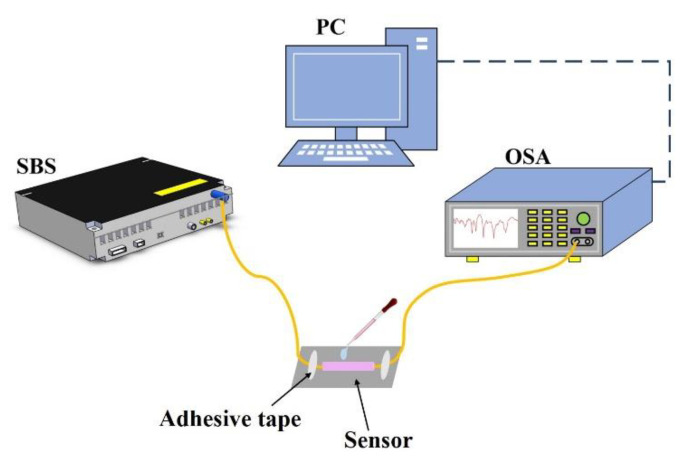
The experimental setup for refractive index measurement.

Figure 5 shows the experimental setup for measuring the response of the proposed all-fiber sensor based on the SMF–HCF–FCF–HCF–SMF structure to the variations in RI. The super-continuum broadband light source (SBS, Wuhan Yangtze Soton Laser Co., Ltd., Wuhan, China) with the monitoring light wavelength range of 600–1700 nm is connected to the lead-in SMF end of the proposed sensor, and the continuous light is transmitted through the lead-out SMF end of the sensor to the optical spectrum analyzer (OSA, Yokogawa, Tokyo, Japan) with the wavelength resolution of 0.02 nm for observation. The data obtained is analyzed by a computer (PC).

**Figure 6:**
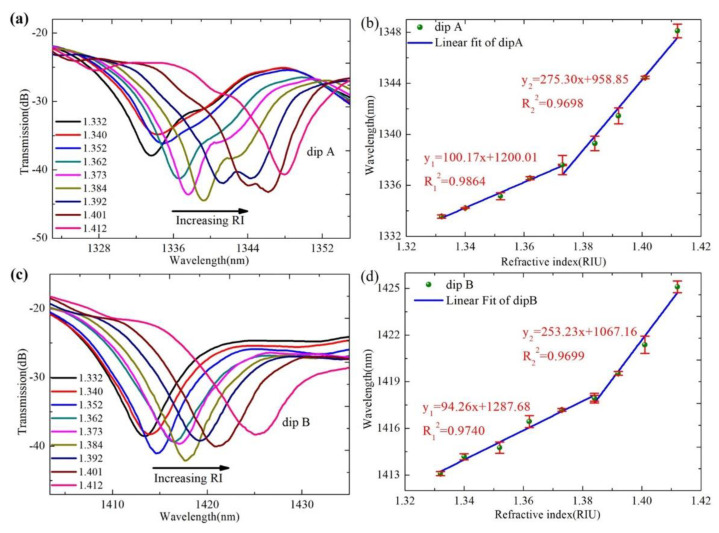
(**a**) The transmission spectra of dip A at different RI; (**b**) the wavelength shift trends of dip A under different RI; (**c**) the transmission spectra of dip B at different RI; and (**d**) the wavelength shift trends of dip B under different RI.

When the room temperature is maintained at 25 °C, the response of the sensor to external refractive index is studied by completely immersing the sensor in different standard solutions. The standard solutions are mixtures of glycerol and water in different proportions, the refractive indices of which are calibrated using an Abbe refractometer. The corresponding RIs are 1.332, 1.340, 1.352, 1.362, 1.373, 1.384, 1.392, 1.401, and 1.412, respectively. For the accuracy of the experiment and to eliminate errors, each RI point is kept for 5 min to ensure sufficient time to stabilize the transmission spectrum. After each measurement, the sensor is cleaned with deionized water and compressed air, and then immersed in a standard solution with an increasingly high RI. Figure 6a,c show the transmission spectra of dip A and dip B at external RIs of 1.332, 1.340, 1.352, 1.362, 1.373, 1.384, 1.392, 1.401, and 1.412, respectively. It can be observed that the wavelengths of the transmission spectra under different RIs change very significantly. When the RI increases from 1.332 to 1.412, the valley wavelength of dip A and dip B gradually shifts to the long wavelength direction. According to Equation (4), we can see that in the case of high refractive index, the difference between the effective refractive index and the external refractive index of the interference mode is greater, and the change in wavelength is also greater. To study the variation trend of wavelength dip more intuitively, Figure 6b,d shows the change trend diagrams of the corresponding wavelengths of dip A and dip B under different RIs. As can be seen from the figures, the sensor is more sensitive in the high refractive index range. Over the RI range from 1.332 to 1.412, the variations of the dip A and dip B are 14.35 nm and 12.24 nm, respectively. The maximum RI sensitivities of the dip A and dip B are 275.30 nm/RIU and 253.23 nm/RIU, respectively.

### 3.3. Temperature Sensing

**Figure 7:**
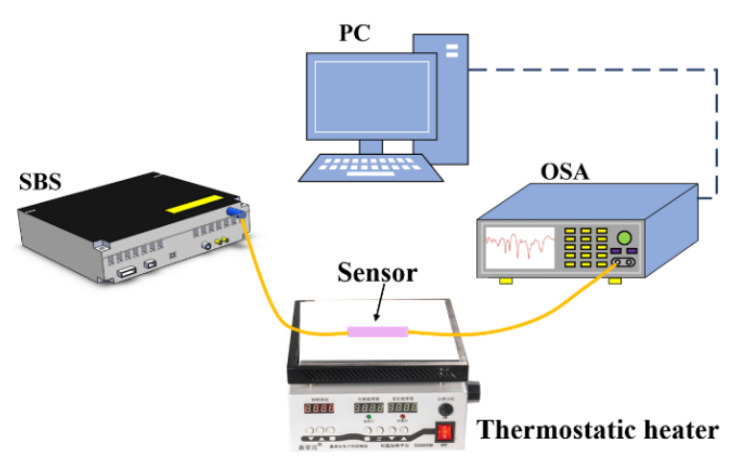
The experimental setup for temperature measurement.

**Figure 8:**
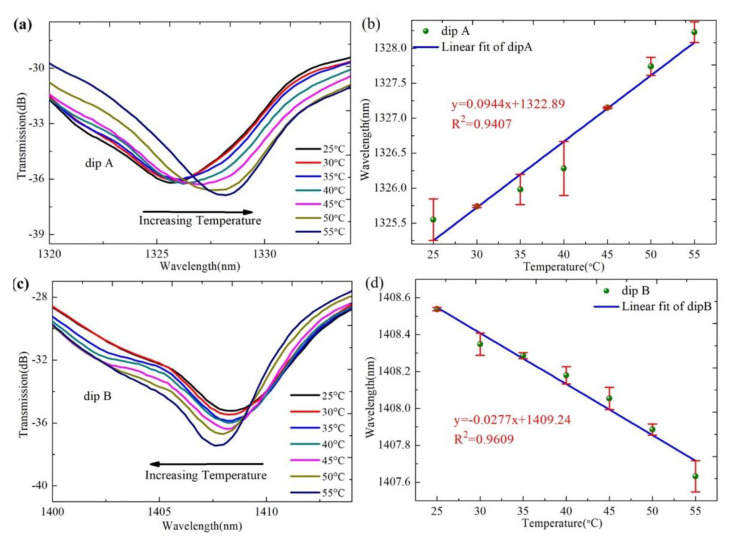
(**a**) The transmission spectra of dip A at different temperature; (**b**) the wavelength shift trends of dip A under different temperature; (**c**) the transmission spectra of dip B at different temperature; and (**d**) the wavelength shift trends of dip B under different temperature.

Next, we analyze the temperature response characteristics of the proposed sensor. The sensor is placed on a thermostatic heater without external axial tension, as shown in Figure 7. The temperature increases from 25 °C to 55 °C with a step of 5 °C by adjusting the thermostatic heater. An optical spectrum analyzer (OSA) is used to record the transmission spectrum of each temperature point. With the increase in temperature, the spectrum of the sensor shifts due to the thermo-optical effect and thermal expansion effects of the four-core fiber. Figure 8a,c show the transmission spectra of dip A and dip B at different temperatures. Obviously, with the increase in temperature, the wavelength of dip A gradually shifts to the long wavelength direction, and the wavelength of dip B gradually shifts to the short wavelength direction. As shown in Figure 8b,d, in the temperature range of 25 °C to 55 °C, the migrations of dip A and dip B are 2.68 nm and 0.91 nm, respectively. It can be seen from the fitting results that the migration is linear. The linear values of temperature response for dip A and dip B are 0.9407 and 0.9609, and the temperature sensitivities are 94.4 pm/°C and –27.7 pm/°C, respectively.

### 3.4. Analysis of Experimental Results

By monitoring the wavelength shift of the two selected dips, RI and temperature can be measured simultaneously. When RI and temperature act on the sensor simultaneously, a matrix equation can be used to distinguish between RI and temperature variations since there are two different interference dips (*dip A* and *dip B*) with different sensitivities to RI and temperature. The wavelength shifts of *dip A* and *dip B* can be represented by the following matrix:

(6) $$\left[\begin{array}{c} \Delta \lambda_{dipA}\\ \Delta \lambda_{dipB} \end{array}\right]=\left[\begin{array}{cc} K_{n,dipA} & K_{T,dipA}\\ K_{n,dipB} & K_{T,dipB} \end{array}\right]\left[\begin{array}{c} \Delta n\\ \Delta T \end{array}\right]$$

where $\Delta \lambda_{dipA}$ and $\Delta \lambda_{dipB}$ are the wavelength shifts of *dip A* and *dip B* in the interference spectrum, respectively; $K_{n,dipA}$ and $K_{T,dipA}$ are respectively the sensitivities of the RI and temperature for *dip A*. Similarly, $K_{n,dipB}$ and $K_{T,dipB}$ are those for *dip B*. Δn and ΔT are the changes in RI and temperature, respectively. Through the inverse matrix operation, it can be obtained:

(7) $$\left[\begin{array}{c} \Delta n\\ \Delta T \end{array}\right]=\frac{1}{M}\left[\begin{array}{cc} K_{T,dipB} & -K_{T,dipA}\\ -K_{n,dipB} & K_{n,dipA} \end{array}\right]\left[\begin{array}{c} \Delta \lambda_{dipA}\\ \Delta \lambda_{dipB} \end{array}\right]$$

where $M=K_{n,dipA}K_{T,dipB}-K_{T,dipA}K_{n,dipB}$. Based on the above experimental results, $K_{n,dipA}=\;275.3$, $K_{T,dipA}=\;0.0944$, $K_{n,dipB}=\;253.23$, and $K_{T,dipB}=\;-0.0277$. Substituted into Equation (7), we can get:

(8) $$\left[\begin{array}{c} \Delta n\\ \Delta T \end{array}\right]=-\frac{1}{31.5307}\left[\begin{array}{cc} -0.0277 & -0.0944\\ -253.23 & 275.3 \end{array}\right]\left[\begin{array}{c} \Delta \lambda_{dipA}\\ \Delta \lambda_{dipB} \end{array}\right]$$

After obtaining the resonant wavelength shifts of *dip A* and *dip B*, the changes of RI and temperature can be calculated using this matrix. The RI resolution and temperature resolution of our proposed sensor are 7.74 × 10^−5^ RIU and 0.335 °C, respectively, when the OSA has a wavelength resolution of 0.02 nm.

To evaluate the sensor proposed in this article, we compare its performance with other MZI sensors which are based on multi-core fiber (MCF), hollow-core fiber (HCF), thin-core fiber (TCF), multimode fiber (MMF), etc. The summary comparison is listed in Table 1. It clearly shows that our sensor can not only achieve the dual-parameter measurement of RI and temperature, but also has better characteristics. For the RI response, the proposed structure can be measured in a wider range; for the temperature response, the sensitivity of this structure is higher.

### 3.5. Repeatability Experiment of Sensor

**Figure 9:**
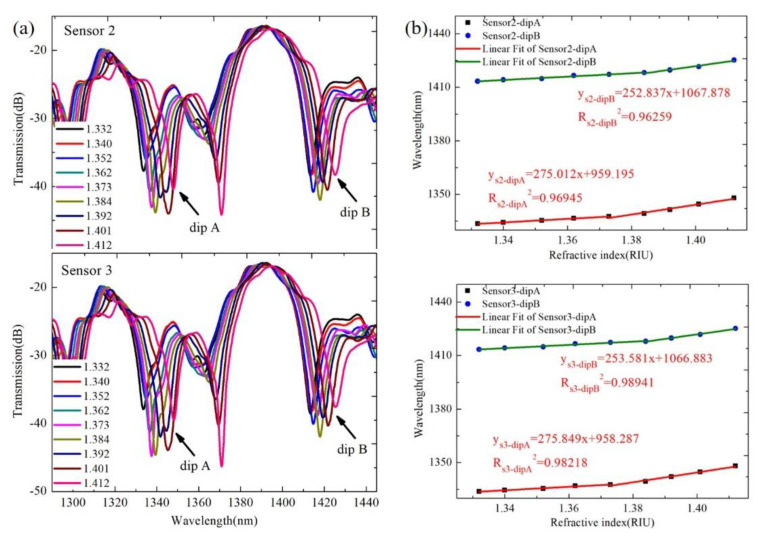
(**a**) The interference spectra of Sensor 2 and Sensor 3 at different RI; (**b**) the wavelength shift trends for Sensor 2 and Sensor 3 under different RI.

**Figure 10:**
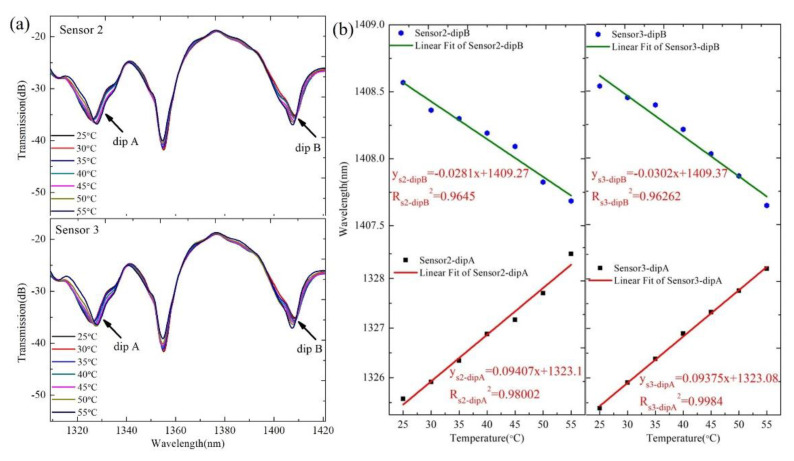
(**a**) The interference spectra of Sensor 2 and Sensor 3 at different temperature; (**b**) the wavelength shift trends for Sensor 2 and Sensor 3 under different temperature.

In order to explore the reproducibility of the proposed sensor based on the SMF–HCF–FCF–HCF–SMF structure, we fabricated two new sensors (Sensor 2 and Sensor 3) with identical parameters and performed the same refractive index and temperature sensing experiments. Then, according to the same data processing method as described above, the transmission spectra and the change trend diagrams of the corresponding wavelengths for the repeated experiments are obtained, as shown in Figure 9 and Figure 10.

From Figure 9b and Figure 10b, it can be seen that for Sensor 2, the maximum RI sensitivities of dip A and dip B are 275.012 nm/RIU and 252.837 nm/RIU, respectively, and the temperature sensitivities are 94.07 pm/°C and –28.1 pm/°C, respectively. The maximum RI sensitivities of dip A and dip B of Sensor 3 are 275.849 nm/RIU and 253.581 nm/RIU, and the temperature sensitivities are 93.75 pm/°C and –30.2 pm/°C, respectively. By comparing the RI and temperature sensitivity of the three sensors, we can see that they are very approximate, so our proposed sensor based on the SMF–HCF–FCF–HCF–SMF structure has satisfactory repeatability.

## 4. Conclusions

In conclusion, we propose and demonstrate an all-fiber m-MZI sensor based on the SMF–HCF–FCF–HCF–SMF structure. The two interference dips of the sensor have different sensitivities to RI and temperature, thus allowing a simple and fast demodulation based on the sensitivity matrix for simultaneous measurement of RI and temperature. The experimental results show that the proposed sensor has a maximum RI sensitivity of 275.30 nm/RIU from 1.332 to 1.412 and a maximum temperature sensitivity of 94.4 pm/°C in the temperature range of 25 °C to 55 °C. Compared with other sensors, the sensor has a simple fabrication process because it involves only fusion, and has good repeatability on the premise of ensuring cutting accuracy. At the same time, it has the advantages of compact structure, wide measurement range, and the ability to measure RI and temperature simultaneously. In addition, due to the limitation of the low thermo-optical coefficient of optical fiber materials, to further improve the temperature sensitivity, the temperature response performance of the sensor can be enhanced by filling the core of hollow-core fiber with high thermal sensitive materials such as polydimethylsiloxane (PDMS). The performance of the designed fiber sensor can also be improved by optimizing the structure parameters after a comprehensive simulation (e.g., the optical field mode and its transmission in the optical fiber, the optical fiber fusion loss, etc.) using an advanced photonic modeling and simulation tool.

## Figures and Tables

**Table 1:** Comparison with other MZI sensors of similar structure.

Sensor Structure	Sensitivity of RI	Sensitivity of Temperature	Simultaneous Detection	Ref.
MMF–eMCF–MMF + FBG	−11 nm/RIU at 1.34–1.38	56.42 pm/°C at 24–130 °C	no	[36]
SMF–TFCF–SMF	171.2 nm/RIU at 1.3448–1.3774	–	no	[37]
MMF–TFCF–MMF	–	0.284 dB/°C at 45–80 °C	no	[38]
SMF–FMF–SCF–SMF	–	91.8 pm/°C at 25.8–108.5 °C	no	[19]
MCF + TFBG	−74.2 dB/RIU at 1.31–1.39	9.75 pm/°C at 10–40 °C	no	[39]
MMF–eMCF–MMF	178.20 dB/RIU at 1.334–1.370	66.73 pm/°C at 30–80 °C	no	[40]
MMF–TCF–MMF + FGB	−0.541 nm/RIU at 1.3479–1.3775	12.45 pm/°C at 30–110 °C	yes	[41]
SMF–HST–SMF	16.76 nm/RIU at 1.333–1.3818	22.58 pm/°C at 20.1–60 °C	yes	[42]
SMF–MMF–SMF + FBG	−41.92 nm/RIU at 1.335–1.372	0.093 nm/°C at 20–60 °C	yes	[43]
SMF–FMF–SMF	−39.15 nm/RIU at 1.336–1.38	0.05 nm/°C at 25–70 °C	yes	[44]
SMF–THCF–SMF	214.97 nm/RIU at 1.333–1.379	2.96 pm/°C at 50–85 °C	yes	[45]
HCF–FCF–HCF	275.30 nm/RIU at 1.332–1.412	94.4 pm/°C at 25–55 °C	yes	This work

## Data Availability

Data available upon request.
